# Instant and short-term effects of acupuncture for depression and anxiety in unstable angina pectoris patients with percutaneous coronary interventions

**DOI:** 10.3389/fcvm.2024.1173080

**Published:** 2024-01-19

**Authors:** Juan Hui Pei, Feng Gan, Yun He Bai, Yan Lin Xing, Jian Jun Jia, Huan Wang

**Affiliations:** ^1^ Department of Cardiovascular Medicine, Beijing Aerospace General Hospital, Beijing, China; ^2^ Institute of Geriatrics, Chinese People’s Liberation Army General Hospital, Beijing, China; ^3^ The Second Medical Center, Chinese PLA General Hospital, Beijing, China

**Keywords:** coronary heart disease, heart rate variability, cardiovascular autonomic function, acupuncture, depression

## Abstract

**Aim:**

Patients with unstable angina pectoris (UAP) usually present anxiety or depression during percutaneous coronary intervention (PCI). This study sought to investigate the instant and short-term effects of acupuncture for anxiety and depression in UAP patients with PCI.

**Methods:**

A total of 210 UAP patients who underwent PCI were recruited and randomly assigned (1:1:1) to acupuncture, placebo, or control groups. Enzyme-linked immunosorbent assay was used to detect the levels of fasting glucose, fasting insulin, homeostasis model assessment of insulin resistance (HOMA-IR), interleukin-6 (IL-6), high-sensitivity C-reactive protein (Hs-CRP), advanced oxidation protein products (AoPPs), and oxidized low-density lipoprotein (OX-LDL). Serial questionnaires with the Hamilton Anxiety (HAMA) scale and the Pittsburgh Sleep Quality Index were evaluated, and heart rate variability indicators were obtained.

**Results:**

Primary end-point: low frequency/high frequency (HF) was lower in the electroacupuncture group (*p* = 0.014), while standard deviation of normal-to-normal intervals, average standard deviation of normal-to-normal intervals, percentage of successive intervals that differ more than 50 ms, and HF were increased with acupuncture (*p* = 0.018, *p* = 0.043, *p* = 0.016, and *p* = 0.002, respectively). Secondary end-point: significant improvements in anxiety levels (HAMA) were observed in the three groups (*p* < 0.001). The fasting insulin and HOMA-IR levels were similar between the control group and the acupuncture group (*p* = 0.285 and *p* = 0.165, respectively). The levels of IL-6 and AoPPs differed among the three groups (*p* = 0.021 and *p* < 0.001, respectively). However, no significant differences were found in fasting plasma glucose, fasting c-peptide, Hs-CRP, and OX-LDL levels among the three groups (*p* = 0.585, *p* = 0.611, *p* = 0.902, and *p* = 0.756, respectively).

**Conclusions:**

In this study, short-term acupuncture may potentially relieve clinical symptoms before PCI treatment.

**Clinical Trial Registration:**

ClinicalTrials.gov, identifier (NCT03789344).

## Introduction

Coronary heart disease (CHD) is one of the leading causes of death. Patients with severe CHD are likely to suffer from mental disorders and are required to receive PCI and take several kinds of medications for their whole life (1, 2). A higher prevalence of mental diseases including depression symptoms and memory disorders in patients with CHD has been demonstrated (3–5). Despite compelling reasons for treating anxiety in patients with CHD, few studies have reported the effective strategy and evaluated the effects of treating anxiety during percutaneous coronary intervention (PCI).

Traditionally, acupuncture is widely used as a complementary therapy in ancient China (6). Acupuncture stimulates acupoints of the body by penetrating the skin with metal needles (7). Evidence has emerged that acupuncture can treat stable angina pectoris, alleviating symptoms, reducing anginal attacks, and decreasing nitroglycerin use (8). Animal studies have shown that acupuncture can protect the ischemic myocardium and improve cardiac function (9–11). Angina pectoris may be mediated by coronary chemoreceptors and transmitted to the brain through vagal afferent nerves. The central nervous system may serve as an essential node of acupuncture treatment for moderate coronary artery lesions with stable angina pectoris. Heart rate variability (HRV) is an index of the balance between the sympathetic and parasympathetic nervous systems, and it is used to monitor cardiac vagal function (12, 13). In our previous research, we applied continuous auricular electroacupuncture measurements in patients with depression using computer-based HRV recordings before, during, and after long-lasting electroacupuncture. This study documented that auricular acupuncture significantly improved various aspects of quality of life and also highlighted the significant increase in HRV (14). Our animal experiment demonstrated that needling the acupuncture Neiguan point can significantly improve the HRV of beagles with low HRV levels induced by atropine. The data mainly suggest that acupuncture can mediate sympathetic nerve function after vagotomy (15). However, the vagus nerve's function is to protect the heart (16).

The abovementioned studies have shown that acupuncture can improve HRV, protect the heart, and reduce anxiety. The main goal of the present study is regarding the effects of acupuncture performed during perioperative PCI on patients who have both heart problems and depression. Compared with drug treatment, acupuncture has no side effects and no dependence. In the present study, we carried out a randomized controlled trial to investigate the instant and short-term effects of acupuncture on UAP patients with anxiety and depression during PCI treatment.

## Materials and methods

### Patients

This was an open-label, prospective, parallel, randomized, controlled trial on the instant and short-term effects of acupuncture for treating anxiety and depression in UAP patients with PCI. A total of 210 patients diagnosed with unstable angina pectoris and requiring PCI were recruited from Beijing Aerospace General Hospital between June 2021 and August 2022. The clinical diagnosis of UAP is according to the classification criteria of the Chinese Society of Cardiology of Chinese Medical Association (17). This protocol was approved by the ethics committee affiliated with the People's Liberation Army General Hospital (ethical approval number: S2018-070-02; Clinical Trials.gov ID: NCT03789344). All patients provided written informed consent. Patients were randomly assigned (1:1:1) to the electroacupuncture, placebo, or control group by the randomization schedules generated with SAS software. The patients’ screening sequence numbers were printed outside, and the randomization numbers were sealed in opaque envelopes. All envelopes were numbered consecutively. One researcher screened the eligible patients after baseline, opened the envelopes, and assigned the patients to either the electroacupuncture group, placebo group, or the control group. The electroacupuncture group received electroacupuncture, the control group received flupentixol melitracen tablets, and the placebo group received only simple touch on thumbs without hand reflexology or acupressure stimulation. HRV measurements were completed on the first and third days of admission. The study concluded with a safety follow-up period lasting more than 3 days.

### Inclusion and exclusion criteria

Key inclusion criteria were patients (aged ≥18 years) without routine use of anxiolytic drugs, not pregnant, devoid of a history of drug addiction, lacking a cardiac pacemaker, free from sensory impairments, and no history of reflexology massage. Key exclusion criteria included acute coronary syndrome (ACS) with emergency PCI, congenital heart disease, myocarditis, conduction block, severe cardiopulmonary insufficiency, severe liver and kidney insufficiency, peripheral vascular diseases, infections, autoimmune diseases, malignant tumors, serious systemic diseases, and history of mental illness, cognitive dysfunction and inability to cooperate with psychological testing, surgery, and trauma.

### Sample size estimation

According to the calculation formula of sample size, the total effective rate of UA is 95%, in combination with previous clinical study information. Consequently, the test is set to a = 0.05, beta = 0.10, using a unilateral test, the initial estimated sample number was 71, and the integer number was. According to the test funds and other related issues, the drop-out rate was limited to <10%. The number of cases in each group was set at 78, and the total number of cases in the three groups was 234. Due to the impact of COVID-19, we included 210 patients (Page 18).

### Laboratory methods

The enzyme-linked immunosorbent assay (ELISA) kit (FU-R1770) was applied to detect the serum levels of fasting glucose, glycated hemoglobin, fasting insulin, C-peptide, homeostasis model assessment of insulin resistance (HOMA-IR), interleukin-6 (IL-6), high-sensitivity C-reactive protein (Hs-CRP), advanced oxidation protein product (AoPPs), and oxidized low-density lipoprotein (OX-LDL).

### Electroacupuncture—points and treatment

**Figure 1:**
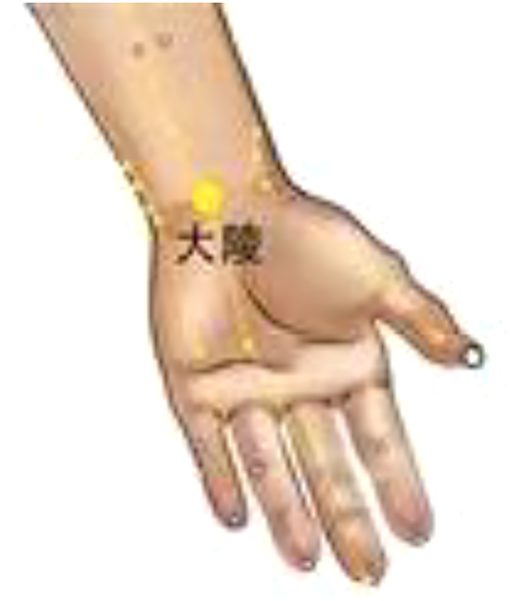
Daling (PC7).

**Figure 2:**
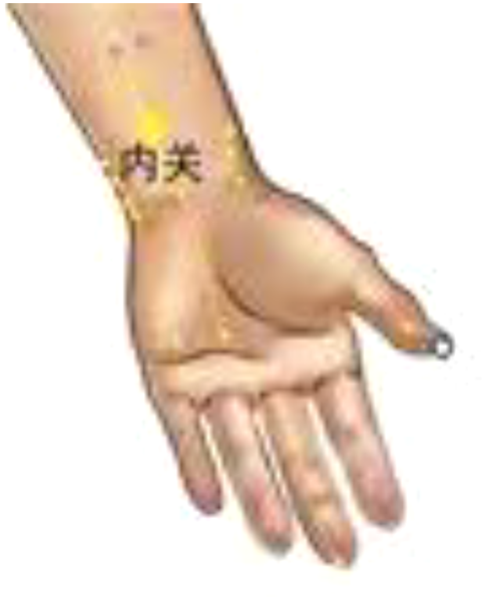
Neiguan (PC6).

**Figure 3:**
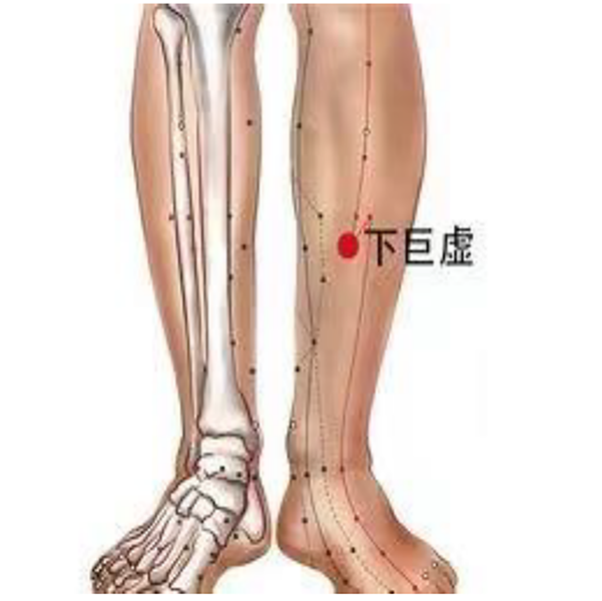
Xiajuxu (ST39).

Daling (PC7) and Neiguan (PC6) are commonly used to treat heart disease, and we applied PC6 stimulation on beagles to improve the HRV. Xiajuxu (ST39) is the lower He Sea acupoint of the small intestine meridian. However, the small intestine meridian and heart meridian exhibit exterior–interior relationships. That is why ST39 is often used clinically to treat heart-related diseases. Daling (PC7), Neiguan (PC6), and Xiajuxu (ST39) were selected as electroacupuncture points. The Daling acupoint (Figure 1) is an acupoint of the palm bag and meridians, in the midpoint of the wrist and palm stripes of the human body, between the palmaris longus tendon and the flexor carpi radialis tendon, and is characterized by straight thorns, measuring 0.3–0.5 in. The Neiguan point is one of the common acupoints on the pericardium meridian of the hand Juyin, and it is located on the palmar side of the forearm, on the line between Quze and Daling, 2 in. above the wrist transverse lines, between the palmaris longus tendon and the flexor carpi radialis tendon. This point is often used for treating angina pectoris, myocarditis, arrhythmia, gastritis, hysteria, and so on; it is punctured straight to a depth of 0.5–1 inch (Figure 2). The Xiajuxu point is located on the anterolateral side of the human leg when the Dubi point is 9 in. from the tibial front edge of a horizontal finger (middle finger) (Figure 3). All three sites were treated with electroacupuncture. The SZ-II electronic low-frequency (LF) acupuncture instrument, manufactured by Suzhou Medical Supplies Factory Co. Ltd. and the Hua Tuo brand, was selected. The waveform was an asymmetric bidirectional pulse wave, and the density wave was adopted. The frequency was 2/100 Hz, the wave width was 0.2 ms, and the intensity was 10 mA. Each treatment session lasted for 30 min, once a day for 3 days.

### Assessment of anxiety, depression, and sleep

According to CCMD-3 Chinese diagnostic criteria for mental disorders, the Hamilton Anxiety (HAMA) scale consists of 14 projects (rated 0, 1, 2, 3, and 4) related to anxiety (18). The Hamilton Depression (HAMD) scale is a semi-structured diagnostic interview evaluating depression, and higher scores on the scale correspond to a worse state of subjective depression. Subjective sleep quality was assessed using the Pittsburgh Sleep Quality Index (PSQI) and self-reported sleep diary. The total score of the PSQI ranged from 0 to 21, with higher scores indicating lower subjective quality of sleep.

### Heart rate variability

This study used time and frequency domains to measure autonomic nervous system (ANS) activity. Time domain measures were evaluated by examining the intervals between heartbeats or normal-to-normal (NN) segments measured in milliseconds, including standard deviation of normal-to-normal (SDNN) interval, average standard deviation of normal-to-normal (SDANN) interval, root mean square of successive differences (RMSSD), and percentage of successive intervals that differ more than 50 ms (PNN50). Spectral analyses of fluctuations in HR yielded frequency domain HRV variables. These included HF peak power between 0.15 and 0.40 Hz and LF peak power between 0.04 and 0.15 Hz. HF power reflects ventilatory modulation of R-R intervals (respiratory sinus arrhythmia) with the efferent impulses on the cardiac vagus nerves, and the LF range is associated with sympathetic nervous system (SNS) activity; the parasympathetic nervous system (PNS) is represented by the high-frequency (HF) range. The LF/HF ratio is helpful for the measurement of ANS activity, as higher LF/HF ratios possibly reflect high SNS activity.

### Statistical analysis

Continuous values were expressed as mean ± standard deviation (SD) and compared with Student's *t*-test, Mann–Whitney *U* test, or one-way analysis of variance (ANOVA) where appropriate. The chi-squared test was used to analyze categorical variables. Analysis of variance was conducted to compare the differences (discharge index − hospital index) and assess whether there is a difference among treatment groups. A *p*-value < 0.05 was considered statistically significant. SPSS 23.0 software (SPSS Inc., Chicago, IL, USA) and SAS 9.4 software were used in this study.

## Results

### Patient baseline characteristics

Hypertension, hyperlipidemia, cardiac insufficiency, and the number of diseased and treated vessels were analyzed in 210 UAP patients (70 patients in each group) undergoing PCI. Patients’ baseline characteristics are summarized in Table 1. No significant differences in these characteristics were found among the three groups (*p *> 0.05, respectively).

**Table 1:** Comparison of characteristics of patients in the three groups.

Group characteristics	Electroacupuncture group (*n* = 70)	Control group (*n* = 70)	Placebo group (*n* = 70)	*p*
Age (years)	66.31 ± 13.74	63.37 ± 11.69	65.70 ± 11.65	0.336
Sex				
Male	48 (68.57%)	47 (67.14%)	45 (64.29%)	0.861
Female	22 (31.43%)	23 (32.86%)	25 (35.71%)	
Hypertension	40 (57.14%)	39 (55.71%)	38 (54.29%)	0.944
Hyperlipidemia	47 (67.14%)	45 (64.29%)	46 (65.71%)	0.939
Cardiac insufficiency				
No	24 (34.29%)	31 (44.29%)	32 (45.71%)	0.826
HfrEF	9 (12.86%)	9 (12.86%)	8 (11.43%)	
HfmdEF	19 (27.14%)	15 (21.43%)	13 (18.57%)	
HfpEF	18 (25.71%)	15 (21.43%)	17 (24.29%)	
Number of diseased vessels				
Single lesions	10 (14.3%)	9 (12.9%)	12 (17.1%)	0.617
Double lesions	24 (34.3%)	28 (40%)	31 (44.3%)	
Three lesions	36 (51.4%)	33 (47.1%)	27 (38.6%)	
Number of treated vessels				
Single vessel	59 (84.3%)	63 (90%)	63 (90%)	0.484
Double vessel	11 (15.7%)	7 (10%)	7 (10%)	

HfrEF, heart failure with reduced ejection fraction; HFmdEF, heart failure with mid-range ejection fraction; HFpEF, heart failure with preserved ejection fraction.

### Effectiveness of short-term acupuncture on primary outcome measure HRV

HRV indicators showed that HR after treatment decreased significantly, but SDNN, SDANN, PNN50, and HF levels were significantly elevated after treatment in all three groups (*p *< 0.05, respectively). In the acupuncture group, the LF/HF ratio decreased significantly compared to before acupuncture, and there was a statistical difference. There was no change in other indexes before and after treatment (Table 2).

**Table 2:** HRV before and after treatment on Days 1 and 3 in the three groups.

	Electroacupuncture group				Control group				Placebo group			
	Day 1	Day 3	Day 1–Day 3	*p*	Day 1	Day 3	Day 1–Day 3	*p*	Day 1	Day 3	Day 1–Day 3	*p*
HR	67.8 ± 2.09	64.9 ± 1.62	2.9 ± 2.5	0.372	67.9 ± 2.09	64.79 ± 1.85	3.11 ± 2.77	0.009^a^	67.77 ± 2.11	64.87 ± 1.67	2.9 ± 2.67	0.223
PNN50	4.82 ± 10	6.32 ± 11.08	−1.5 ± 5.05	0.016^a^	4.29 ± 9.95	6.64 ± 12	−2.36 ± 6.45	0.003^a^	4.29 ± 9.94	6.28 ± 11.64	−1.99 ± 5.65	0.004^a^
SDNN	45.86 ± 26.07	77.2 ± 108.77	−31.34 ± 107.93	0.018^a^	46.31 ± 24.28	89.09 ± 132.87	−42.77 ± 133.18	0.009^a^	44.7 ± 24.38	94.94 ± 148.86	−50.16 ± 149.08	0.006^a^
SDANN	29.4 ± 24.04	50.86 ± 79.6	−21.46 ± 83.18	0.034^a^	30.26 ± 22.11	51.27 ± 70.11	−21.01 ± 74.52	0.021^a^	26.59 ± 21.06	67.09 ± 107.3	−40.5 ± 110.78	0.003^a^
RMSSD	34.6 ± 33.44	56.26 ± 126.7	−21.66 ± 124.35	0.150	30.11 ± 26.38	66.21 ± 156.22	−36.1 ± 154.88	0.055	29.57 ± 25.98	75 ± 173.54	−45.43 ± 171.82	0.030^a^
HF	126.84 ± 233.17	170.5 ± 268	−43.65 ± 110.65	0.002^a^	175.46 ± 317.55	232.83 ± 355.22	−57.36 ± 146.71	0.002^a^	172.41 ± 318.18	225.48 ± 356.32	−53.08 ± 145.11	0.003^a^
LF	292.76 ± 1428.79	293.49 ± 1315.71	−0.73 ± 135.79	0.964	482.71 ± 2001.36	484.25 ± 1840.47	−1.54 ± 188	0.946	467.35 ± 2003.83	429.64 ± 1611.99	37.71 ± 496.18	0.527
LF/HF	1.99 ± 1.88	1.79 ± 1.78	0.2 ± 0.68	0.014^a^	1.71 ± 1.55	1.57 ± 1.44	0.14 ± 0.64	0.072	1.59 ± 1.58	1.51 ± 1.38	0.08 ± 0.68	0.315

Day 1 refers to the index value on the first day of admission. Day 3 refers to the index value on the third day of discharge.

^a^ Indicates that there is a statistical difference.

### Effectiveness of short-term acupuncture on the secondary outcome anxiety scale

Severity was evaluated according to different scores on the scale, with numbers 1–5 representing a gradual increase in severity; all admitted patients scored 2 and 3 on the three scales. Following treatment, patients in the three groups experienced relieved anxiety levels, as measured by the HAMA and PSQI scales. However, there was no significant difference in self-control scores on the HAMD scale among the three groups (Table 3). Scale score composition ratios among groups at admission and discharge were compared, and the results showed no statistical difference between HAMA and PSQI. However, the depression status of each group was consistent, so statistical analysis could not be carried out (Table 4).

**Table 3:** Anxiety levels before and after PCI in the three groups.

Scale group	HAMA scale (N)			HAMD scale (N)			PSQI scale (N)		
	Before	After	*p*	Before	After	*p*	Before	After	*p*
Electroacupuncture group									
2	40	58	<0.001	70	70	NS	24	64	<0.001
3	30	12		NS	NS		46	6	
Control group									
2	45	62	<0.001	70	70	NS	21	62	<0.001
3	25	8		NS	NS		49	8	
Placebo group									
2	42	61	<0.001	70	70	NS	22	60	<0.001
3	28	9		NS	NS		48	10	

NS, no significance, *p *> 0.05.

**Table 4:** Summary of scale score composition ratios at admission and discharge in the three groups.

Group scale	Electroacupuncture group		Control group		Placebo group		*p*
	2	3	2	3	2	3	
HAMA							
Before	40	30	45	25	42	28	0.685
After	58	12	62	8	61	9	0.594
HAMD							
Before	70	NS	70	NS	70	NS	NS
After	70	NS	70	NS	70	NS	NS
PSQI							
Before	24	46	21	49	22	48	0.858
After	64	6	62	8	60	10	0.569

NS, no significance.

### Effectiveness of short-term acupuncture on other pre-specified outcome measures

The relationship between metabolic disturbance index, inflammatory response, and oxidative stress products in each group is presented in Table 5 in terms of metabolic disorders. Compared to the control group, fasting insulin and HOMA-IR levels increased in the acupuncture group, but there was no statistical difference. The levels of IL-6 and AoPPs differed among the three groups (*p *< 0.05, respectively). In addition, there were no significant differences in other indicators, such as fasting plasma glucose, fasting c-peptide, Hs-CRP, and OX-LDL, among the three groups (*p *> 0.05).

**Table 5:** Comparison of biological indicators in the three groups.

Group characteristics	Electroacupuncture group (*n* = 70)	Control group (*n* = 70)	Placebo group (*n* = 70)	*p*
Fasting plasma glucose	−0.59 ± 1.82	−0.29 ± 1.59	−0.36 ± 1.7	0.585
Fasting insulin	−0.47 ± 2.3	−0.94 ± 2.86	−1.06 ± 3.33	0.285
Fasting c-peptide	−0.12 ± 0.49	−0.11 ± 0.71	−0.02 ± 0.71	0.611
HOMA-IR	−0.57 ± 2.53	−0.77 ± 2.52	−0.48 ± 1.85	0.165
IL-6	−0.008 ± 0.009	−0.01 ± 0.01	−0.006 ± 0.007	0.021^a^
HS-CRP	−0.01 ± 0.01	−0.01 ± 0.02	−0.01 ± 0.03	0.902
AoPPs	−0.01 ± 0.006	−0.01 ± 0.012	−0.01 ± 0.011	<0.001^a^
OX-LDL	−0.01 ± 0.02	−0.01 ± 0.04	0 ± 0.07	0.756

Values are the mean ± SD or number (%).

^a^ Indicates statistical differences among the three groups, *p *< 0.05.

## Discussion

CHD is the leading cause of death in almost every region of the world. According to the “Report on Cardiovascular Health and Diseases Burden in China: an Updated Summary of 2021” in 2019, cardiovascular diseases accounted for the top cause of death in both urban and rural areas. It is estimated that there are 330 million patients with cardiovascular diseases, including 13 million with stroke and 11.39 million with CHD (19). Mental illness is also a major contributor to the global burden of disease (20). More than 300 million people of all ages live with depression worldwide, and the disease is expected to become the leading cause of worldwide disability by 2030 (21, 22). According to a recent meta-analysis, 14.3% of all deaths worldwide, or approximately 8 million deaths each year, are attributable to mental disorders (23). Compared with the general population, the prevalence of depression is significantly higher in patients with CHD (22), especially in patients with serious coronary artery disease or even PCI.

Acupuncture is a traditional Chinese medicine therapy. Real-world data with long-term follow-up in Taiwan indicated that the benefit of acupuncture intervention was independent of sex, age, comorbidities, and drug use in patients with CHD and that a lower cumulative incidence of CHD was noted in the acupuncture cohort and the benefits are greater over time (24). Rajai et al. reported that the use of acupressure reduced anxiety in patients undergoing coronary angiography (25), and Valiee et al. focused on the effect of acupressure on preoperative anxiety in patients undergoing abdominal surgery, indicating a significant reduction of anxiety compared with placebo (26). It is reported that the cardiocerebral axis and autonomic nervous system are closely related to cardiovascular disease and that acupuncture may adjust the cardiocerebral axis (27). Studies have shown that LF increases and HF decreases in UAP patients due to myocardial ischemia, indicating that sympathetic nerve activity increases and vagal nerve activity decreases at this time (10). The ratio of LF to HF (LF/HF) reflects the cardiac sympathovagal balance, which we believe is an important component of HRV analysis. In addition, studies found that reduced HRV has been observed in patients with mood and anxiety disorders (28). Our study shows that the LF/HF ratio in the acupuncture group decreased significantly after acupuncture treatment. This experimental result demonstrates that acupuncture can balance the vegetative nerve state of patients with CHD by reducing sympathetic nerve activity, which is expected to improve the HRV of patients with perioperative anxiety during PCI and promote cardiac rehabilitation. In addition, the experiment also showed that HR decreased significantly after treatment, but SDNN, average SDANN, PNN50, and HF levels were significantly elevated. HR, SDNN, SDANN, and PNN50 reflect high HRV, which may occur when the relaxation response is activated. The current study might provide additional insight into the impact of improved HRV with acupuncture. The main finding of the present study is that electroacupuncture can effectively improve the HRV, which indicates that acupuncture stimulation has heart-protective and anxiety-relieving effects on UAP patients with PCI.

Our study found that fasting insulin and HOMA-IR both increased in the acupuncture group compared to the control group. Electroacupuncture applied at different frequencies may cause the release of endogenous opioid peptides such as β-endorphin from the adrenal gland and thereby enhance insulin secretion (29). Studies have shown that acupuncture can improve obesity and insulin sensitivity in patients with type 2 diabetes and has a certain therapeutic effect on diabetes (30, 31). Acupuncture at the Zhongwan (CV12) or Zusanli (ST36) acupoint can improve insulin sensitivity or improve glucose tolerance (32, 33). It was previously demonstrated that short-term electroacupuncture (EA) at the Zusanli and Hegu acupoints effectively reduced the dyspeptic symptoms of diabetic gastroparesis (34). Belivani et al. observed decreased fasting blood glucose in obese patients after EA (35).

Studies on the mechanism have focused on increasing neuronal activity and neurotransmitter upregulation (34, 36). However, there are few articles on stimulating Neiguan, Daling, and Xiaju acupoints in patients with coronary heart disease, anxiety, and depression who are undergoing PCI. The mechanism may involve acupuncture treatment to maintain a balance between sympathetic and parasympathetic nerve systems, thereby reducing cardiac oxygen consumption in UAP patients during perioperative PCI; the initial acupuncture, which may cause pain, could lead to elevated levels of inflammatory markers, such as IL-6 and AoPPs.

In summary, our findings support that instant and short-term acupuncture treatment may potentially relieve clinical symptoms before PCI. However, the present study still has limitations. First, the acupuncture sites were limited, and the observation time was short, so there was no observation of the efficacy of multiple sites and long-term acupuncture, which may have led to performance bias. Second, the present study only enrolled a small sample with a short observation time. Third, prospective, multi-center cohort studies can also be conducted in clinical acupuncture.

## Data Availability

The original contributions presented in the study are included in the article/supplementary materials, further inquiries can be directed to the corresponding author.
